# Supplementary material for: Freshwater snail faecal metagenomes reveal environmental reservoirs of antimicrobial resistance genes across two continents
Source: Microb Genom. 2025 Aug 20;11(8):001480. doi: 10.1099/mgen.0.001480 (PMC12367435; doi:10.1099/mgen.0.001480)

Angus M. O’Ferrall, Alexandra Juhász, Sam Jones, Peter Makaula, Gladys Namacha, Shaali Ame, David Oguttu, Aidan Foo, Sekeleghe A. Kayuni, James E. LaCourse, Janelisa Musaya, J. Russell Stothard, Adam P. Roberts

Freshwater snail faecal metagenomes reveal environmental reservoirs of antimicrobial resistance genes across two continents

<u>Supplementary materials:</u>

Supplementary Table 1: Sample characteristics.

Supplementary Table 2: Summary of Illumina sequencing data and metagenome assemblies.

Supplementary Table 3: Correlation between relative abundance of the top 20 bacterial orders and ARG load.

Supplementary Figure 1. Species-level beta-diversity: a) Bray–Curtis dissimilarity matrix; b) Principal coordinates analysis (PCoA) performed using Bray-Curtis distances.

Supplementary Figure 2: Scatter plots showing associations of sequencing depth with: a) ARG detection; b) MAG recovery.

Supplementary Figure 3: Rarefaction curves showing the number of unique ARGs detected following random sub-setting of reads from samples with > 20 million reads following trimming and filtering.

Supplementary Figure 4: Relative abundance of ARGs aggregated by class (RPKM).

Supplementary Figure 5: Read- versus assembly-based ARG detection.

Supplementary Figure 6: Annotation of M5 NODE_173 containing $bla_{OXA-181}$ and ΔlysR, compared to Tn2013 from K. pneumoniae strain KP3 plamid pKP3-A (Accession no. JN205800) containing $bla_{OXA-181}$ flanked by ΔlysR-ΔereA and ISEcp1.

Supplementary Table 1: Sample information and characteristics.

ID	Administrative region	Continent	Site name	Water type	GPS East	GPS South	Snail genus
**M1**	Malawi	Africa	Nkopola	Stream	-14.314	35.144	*Bulinus*
**M2**	Malawi	Africa	Palm Beach	Stagnant	-14.392	35.221	*Bulinus*
**M3**	Malawi	Africa	Mangochi Town	River	-14.452	35.242	*Bulinus*
**M4**	Malawi	Africa	Chipereka	Stagnant	-14.385	35.275	*Bulinus*
**M5**	Malawi	Africa	Mthawira	Stagnant	-16.854	35.302	*Cleopatra*
**M6**	Malawi	Africa	Nsanje Port	Stagnant	-16.932	35.264	*Bulinus*
**Ug1**	Uganda	Africa	Ngamba Island	Lake	-0.106	32.653	*Biomphalaria*
**Ug2**	Uganda	Africa	Kimi Island 1	Lake	-0.085	32.645	*Bulinus*
**Ug3**	Uganda	Africa	Kimi Island 2	Stagnant	-0.088	32.652	*Physella*
**Z1**	Zanzibar	Africa	Bumbwini	Stream	-5.954	39.195	*Bulinus*
**Z2**	Zanzibar	Africa	Donge Mbije	Stagnant	-5.930	39.256	*Pila*
**Z3**	Zanzibar	Africa	Mwera 1	Stagnant	-6.142	39.269	*Cleopatra*
**Z4**	Zanzibar	Africa	Mwera 2	Stagnant	-6.137	39.266	*Lanistes*
**Uk1**	United Kingdom	Europe	Knowsley	Stream	53.437	-2.819	*Anisus*
**Uk2**	United Kingdom	Europe	Preston Montford	Stream	52.724	-2.840	*Lymnea*

Supplementary Table 2: Summary of Illumina sequencing data and metagenome assemblies.

ID	Illumina short reads			Bacterial assembly (contigs ≥ 500 bp)			Proportion of bacterial reads mapping to assembly (%)
	Total read pairs	Read pairs passing QC	Bacterial read pairs after QC	Assembly size (bp)	Total contigs	N50	
**M1**	9,340,457	7,452,698	2,142,223	21,422,957	18,427	1,369	45.79
**M2**	12,257,171	10,083,776	3,342,252	49,476,907	52,617	952	57.66
**M3**	9,982,521	7,954,558	1,905,474	9,999,998	12,847	746	25.24
**M4**	9,629,854	7,647,992	890,800	5,267,659	7,129	712	15.70
**M5**	9,476,943	7,576,553	3,974,033	34,276,162	20,545	2,699	85.05
**M6**	12,399,711	9,917,053	3,993,286	38,011,353	36,921	1,000	70.24
**Ug1**	17,829,973	13,669,537	4,446,415	71,092,332	68,500	1,030	69.58
**Ug2**	18,211,831	14,031,764	5,008,151	67,825,877	65,357	1,081	75.54
**Ug3**	19,554,138	14,801,846	5,708,500	62,394,277	65,605	929	79.15
**Z1**	28,260,918	20,934,433	4,494,974	44,526,232	43,914	1,015	57.69
**Z2**	26,317,771	19,886,506	5,172,786	31,591,691	41,009	738	39.82
**Z3**	33,416,553	24,804,936	8,167,866	59,830,364	68,388	836	55.23
**Z4**	35,991,622	27,329,748	6,982,030	46,160,878	51,450	868	53.83
**Uk1**	31,718,066	25,270,230	15,346,535	97,468,932	93,004	1,075	85.81
**Uk2**	11,924,977	9,191,952	3,141,161	56,224,377	58,668	959	57.51

Supplementary Table 3: Correlation between relative abundance of the top 20 bacterial orders and ARG load.

**Order**	**Mean relative abundance (%)**	**Spearman's correlation (relative abundance vs ARG load)**	
		**Correlation coefficient**	***p*-value**
**Aeromonadales**	9.71	0.357	0.1916
**Bacillales**	2.36	-0.304	0.2708
**Burkholderiales**	16.28	0.086	0.7630
**Caulobacterales**	1.22	0.243	0.3820
**Corynebacteriales**	3.29	-0.439	0.1032
**Deinococcales**	1.09	-0.104	0.7144
**Enterobacterales**	4.79	-0.111	0.6953
**Flavobacteriales**	4.08	0.211	0.4499
**Micrococcales**	3.71	-0.529	0.0454 *
**Micromonosporales**	1.06	-0.654	0.0100 *
**Nostocales**	3.71	-0.711	0.0041 *
**Propionibacteriales**	1.45	-0.664	0.0086 *
**Pseudomonadales**	7.11	0.604	0.0195 *
**Rhizobiales**	10.00	-0.443	0.1002
**Rhodobacterales**	3.72	-0.332	0.2264
**Rhodocyclales**	1.37	-0.075	0.7926
**Rhodospirillales**	1.35	-0.479	0.0735
**Sphingomonadales**	3.00	-0.082	0.7728
**Streptomycetales**	3.22	-0.661	0.0090 *
**Xanthomonadales**	2.74	-0.007	0.9847

** $p < 0.05$ = statistically significant.*

Supplementary Figure 1. Species-level beta-diversity: a) Bray-Curtis dissimilarity heatmap; b) Principal coordinates analysis (PCoA) performed using Bray-Curtis distances.

a

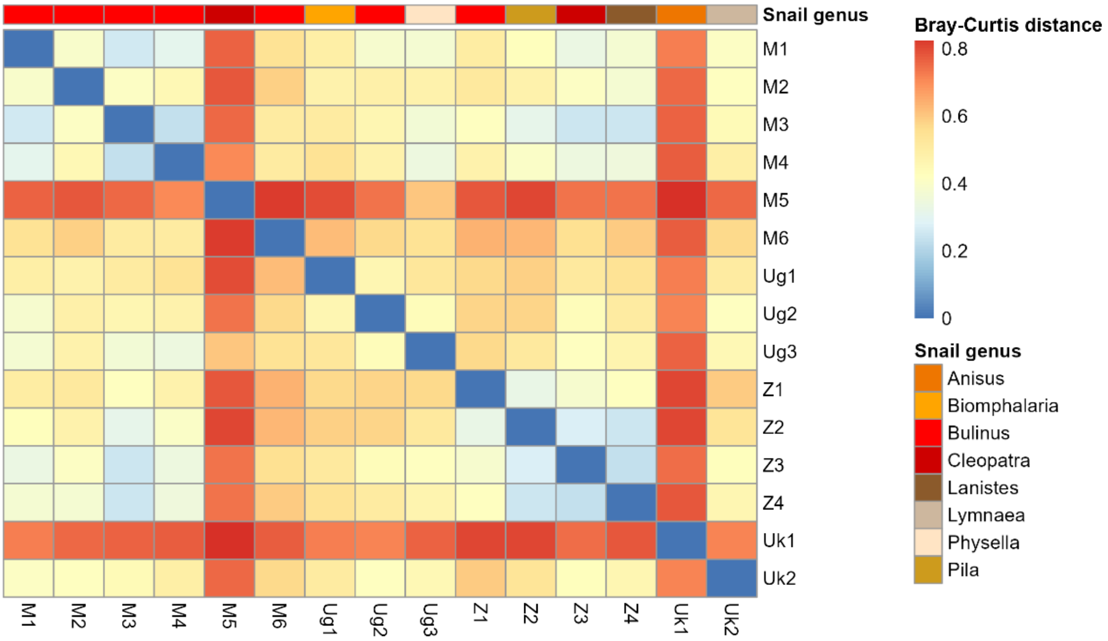

b

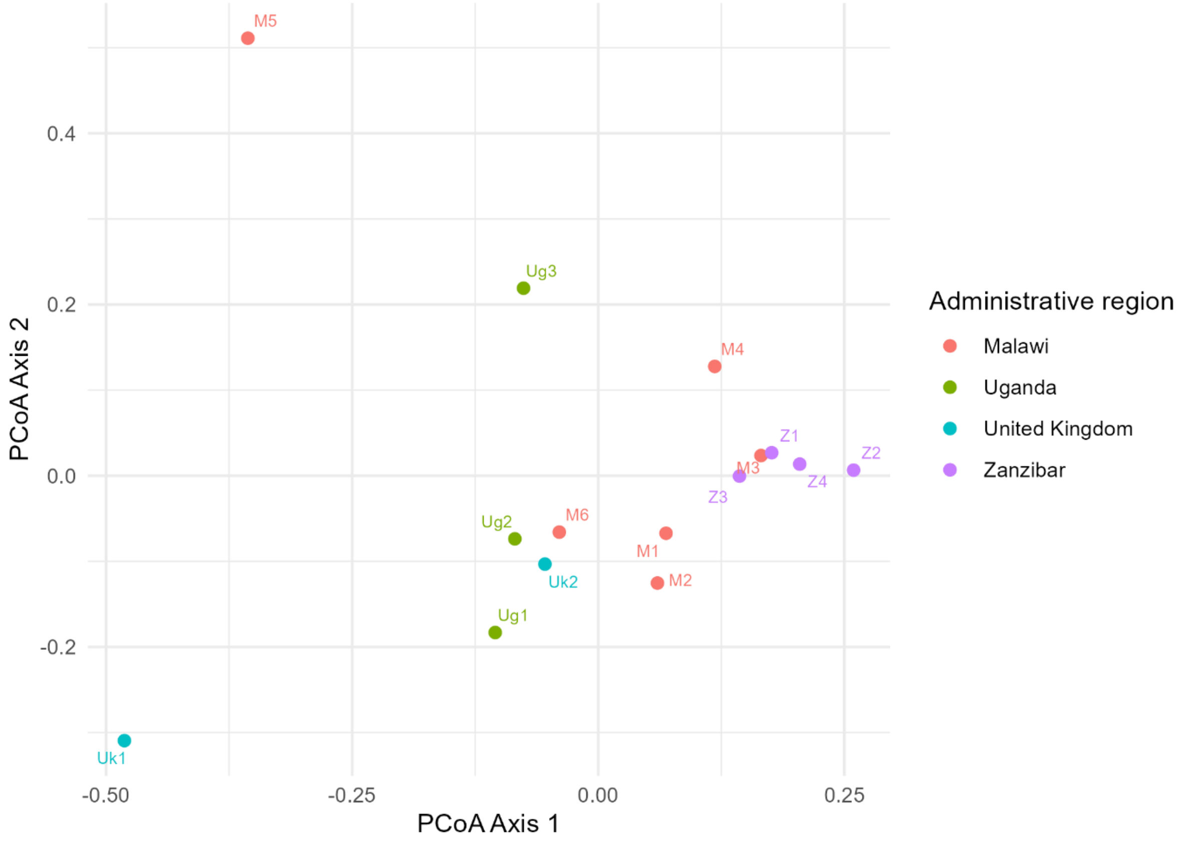

Supplementary Figure 2: Scatter plots showing associations of sequencing depth with: a) ARG detection; b) MAG recovery.

a

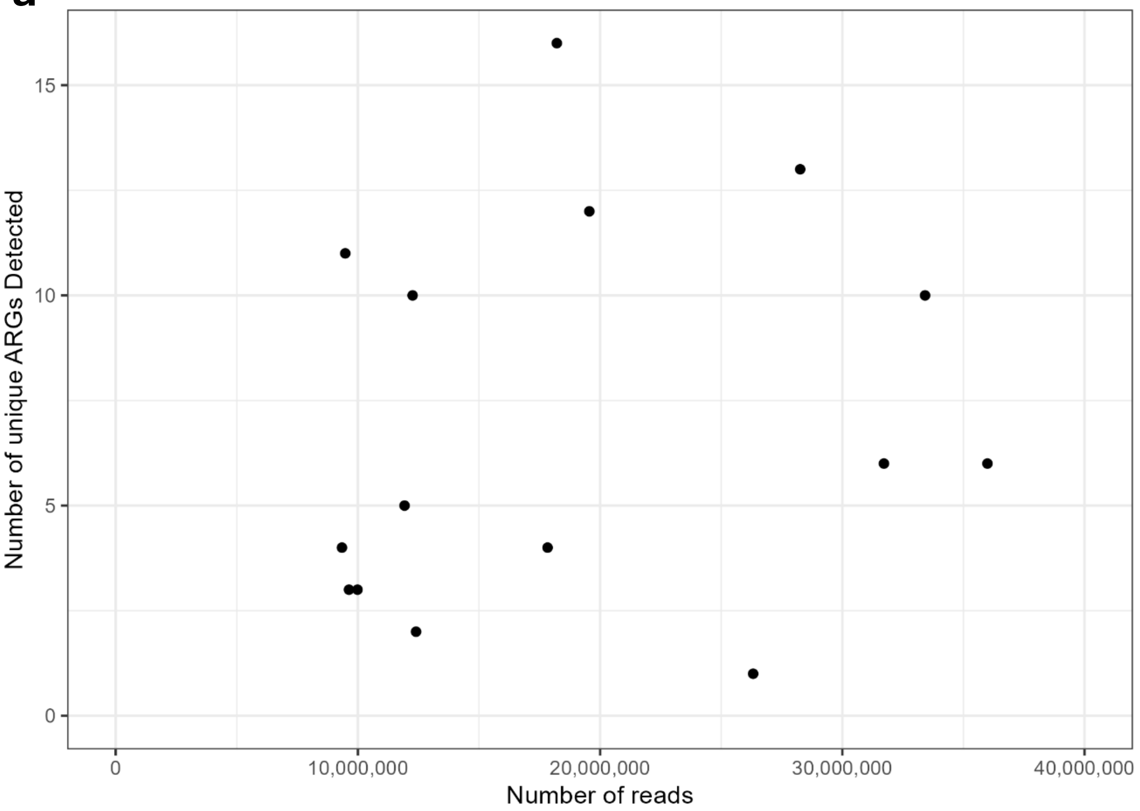

b

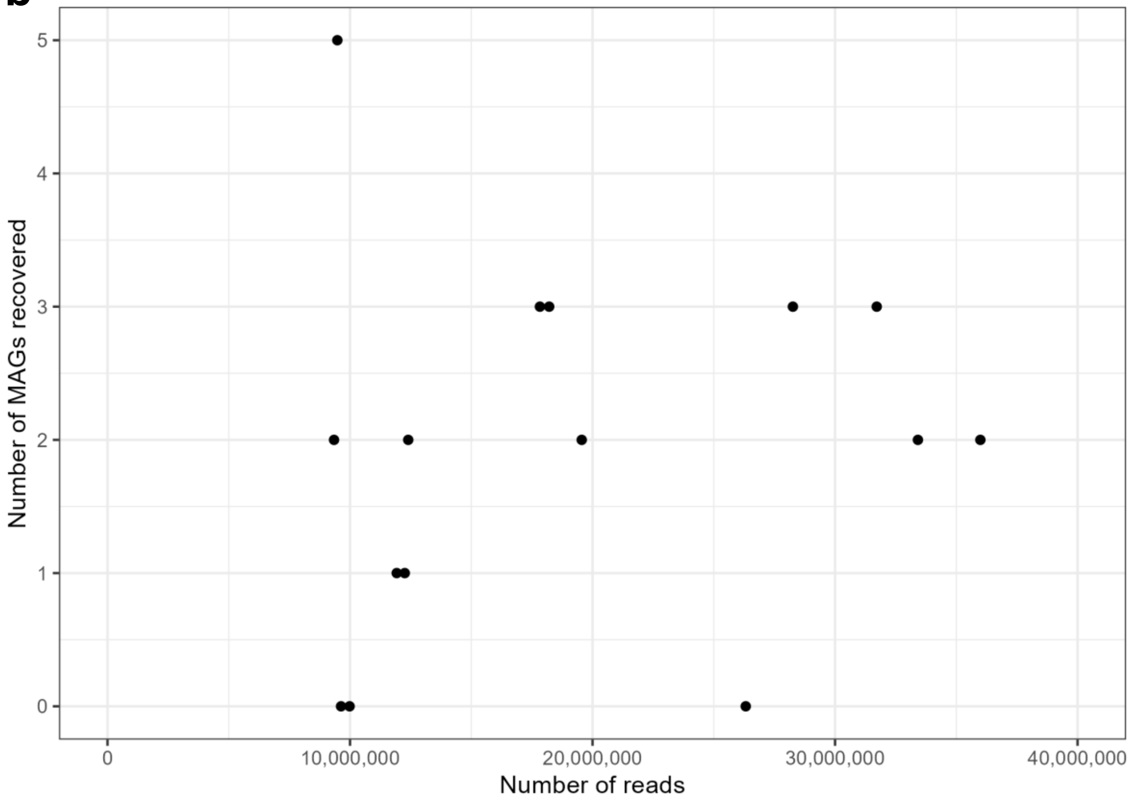

Supplementary Figure 3: Rarefaction curves showing the number of unique ARGs detected following random sub-setting of reads from samples with > 20 million reads following trimming and filtering.

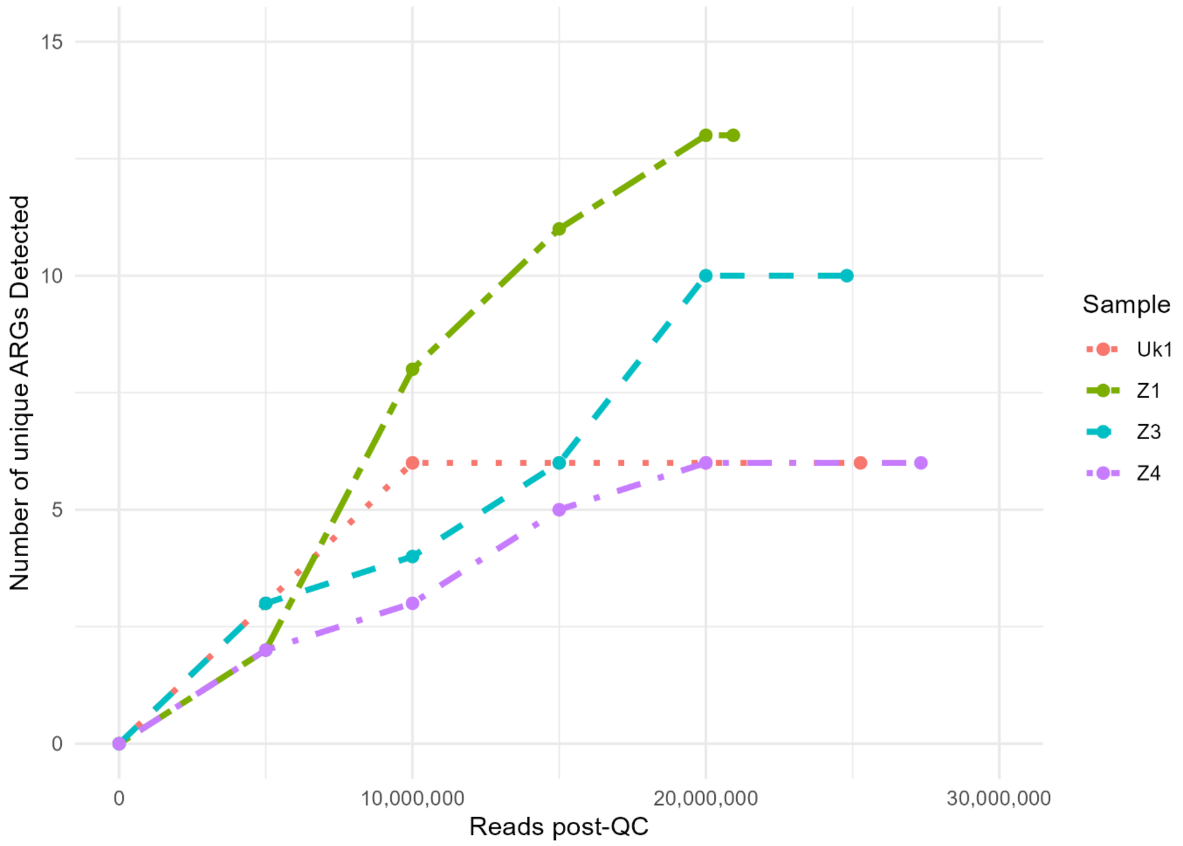

Supplementary Figure 4: Relative abundance of ARGs aggregated by class (RPKM).

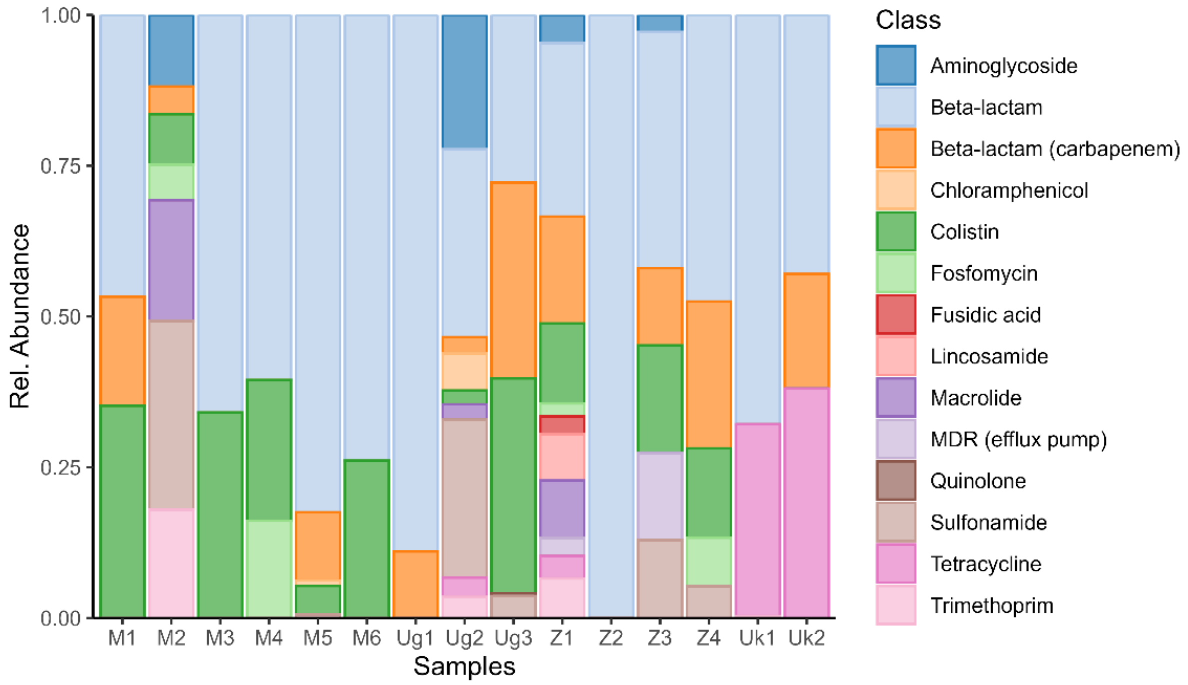

Supplementary Figure 5: Read- versus assembly-based ARG detection.

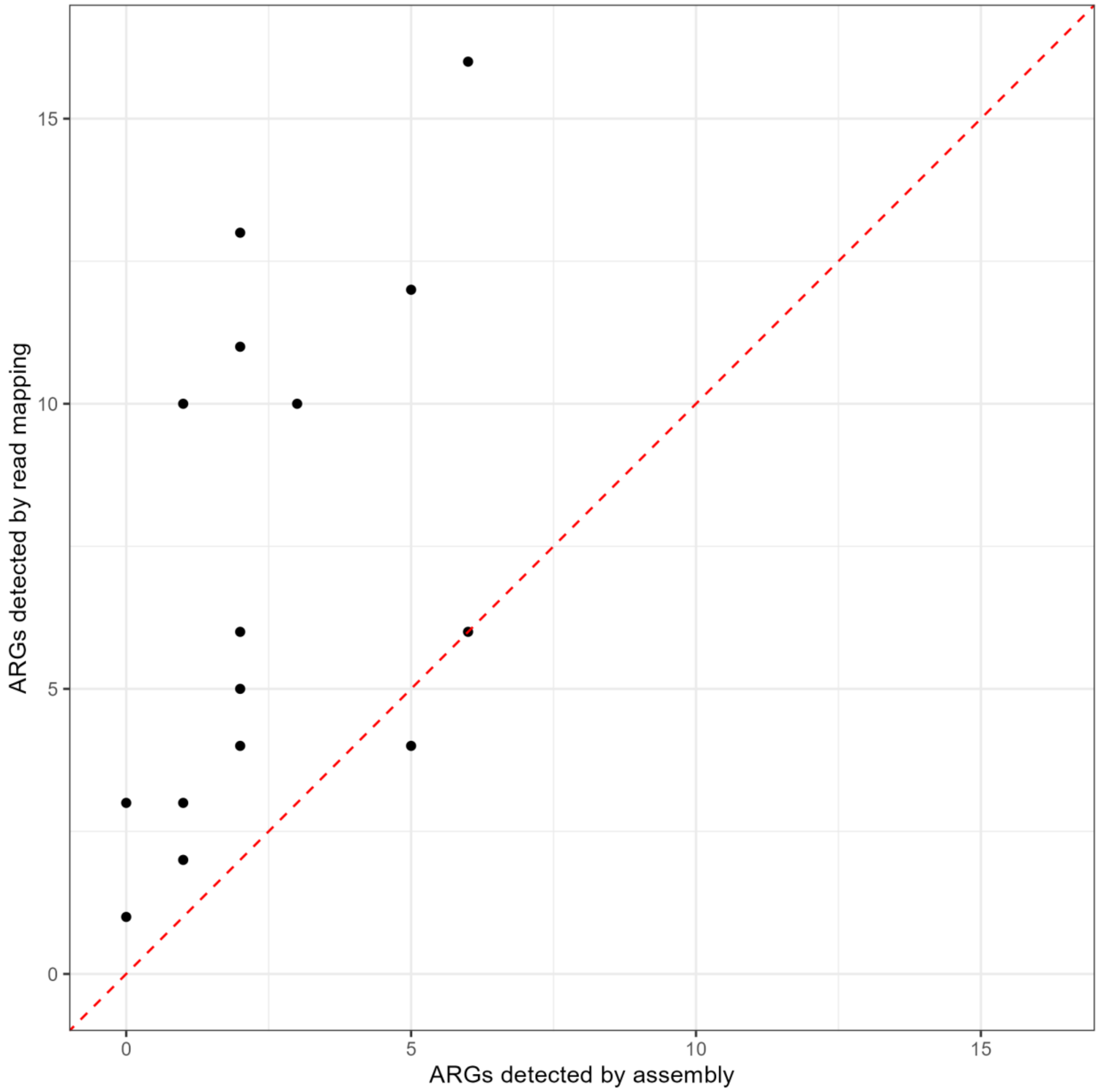

Supplementary Figure 6: Annotation of M5 NODE_173 containing bla$_{OXA-181}$ and ΔlysR, compared to Tn2013 from K. pneumoniae strain KP3 plasmid pKP3-A (Accession no. JN205800) containing bla$_{OXA-181}$ flanked by ΔlysR-ΔereA and ISEcp1.

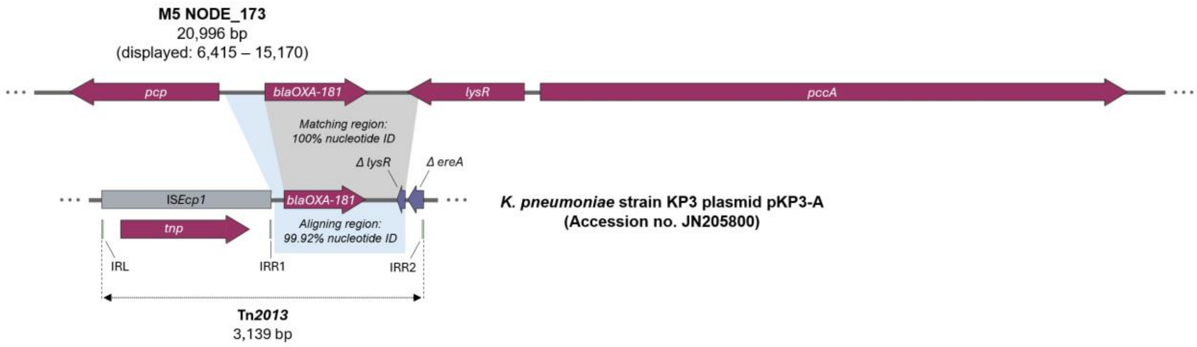

Supplement: Uncited Supplementary Material 1. [file mgen-11-01480-s001.pdf]
